# Benefits of Pilates in Parkinson’s Disease: A Systematic Review and Meta-Analysis

**DOI:** 10.3390/medicina55080476

**Published:** 2019-08-13

**Authors:** David Suárez-Iglesias, Kyle J. Miller, Manuel Seijo-Martínez, Carlos Ayán

**Affiliations:** 1 VALFIS Research Group, Institute of Biomedicine (IBIOMED), Faculty of Physical Activity and Sports Sciences, University of León, 24071 León, Spain; 2 School of Health and Life Sciences, Federation University, Ballarat, Victoria 3353, Australia; 3 Department of Neurology, Complejo Hospitalario Pontevedra-Salnés, 36001 Pontevedra, Spain; 4 Well-Move Research Group, Faculty of Education and Sport Science, Department of Special Didactics, University of Vigo, 36004 Pontevedra, Spain

**Keywords:** parkinson, rehabilitation, pilates, exercise

## Abstract

Pilates may be a beneficial method of exercise for people with Parkinson’s disease (PD). However, no studies have critically reviewed the scientific evidence in this regard. The purpose of this study was to conduct a systematic review and meta-analysis on the effectiveness of Pilates as a rehabilitation strategy for PD. A systematic search of the electronic databases PubMed, PEDro, Scopus, and SPORTDiscus was conducted to identify studies related to the effect of Pilates on PD. The search timeframe ranged from the inception of each database to March 2019. The search resulted in the identification of four randomized controlled trials (RCTs) and four non-RCT studies. The methodological quality of the investigations ranged from poor to fair. The descriptive analysis of the eight investigations showed that Pilates resulted in beneficial effects on fitness, balance and functional autonomy. A subsequent meta-analysis on the four RCTs indicated that Pilates was more effective than traditional training programmes in improving lower limb function. Pilates can be safely prescribed for people with mild-to-moderate PD. Preliminary evidence indicates that its practice could have a positive impact on fitness, balance and physical function. Its benefits on lower-body function appear to be superior to those of other conventional exercises. Future randomized studies with greater samples are needed to confirm these observations.

## 1. Introduction

Parkinson’s disease (PD), the second most common neurodegenerative disease in the world, generates large social and health costs [1]. The impairment caused by PD is largely due to motor dysfunction that progressively limits the functional autonomy of the patient [2]. The medical and surgical therapeutic advances to date have been remarkable, but not definitive, in correcting the symptoms of the disease. For this reason, it is important to evaluate the effects of parallel alternative therapies, among which physical exercise stands out due to its low cost and absence of side effects [3]. Scientific evidence shows that exercise training programmes have a beneficial effect on motor symptoms such as gait, balance, fall risk and physical function [4], as well as non-motor symptoms, including cognitive function, sleep disorders and quality of life [5], in people with PD.

The Spanish national health system is public, universal, accessible, and efficient [6]. However, despite the importance of exercise as a rehabilitation strategy, in the current health system, there are no specific rehabilitation programmes in public hospitals, patient groups, or organizations for people with PD. It has been suggested that neurologists should identify the types of exercises optimal for PD patients and ensure that the methods are safely and effectively implemented [7]. In order to do so, however, neurologists must have up-to-date, detailed, and quality information on the effects of physical exercise on PD. This type of information is generally found by reading published reviews and meta-analyses on the subject.

There are currently several PD reviews that have focused on the feasibility and effectiveness of traditional forms of exercise, such as aerobic and resistance training, as well as that of a more alternative nature, such as martial arts and dance. To date, however, no such study has been published on the practical use of Pilates as a rehabilitation strategy that could improve adherence to exercise in people with PD.

Pilates is a mind–body exercise approach, initially created by Joseph Pilates as a training method for people who were already fit. The original set of exercises has evolved the point that a sound modern interpretation of Pilates can be applied to both fit and unfit people [8]. For rehabilitation purposes, the Pilates method aims to improve posture and control of movement via neuromuscular control techniques, which are believed to improve lumbar spine stability by targeting the local stabilizer muscles of the lumbar-pelvic region [9]. In the field of physical therapy, there are two primary ways of practicing Pilates. The first is “Mat Pilates”, which is focused on the performance of exercises on a mat on the floor. The second, usually known as “Apparatus or Equipment Pilates”, is carried out by means of a resistant apparatus or barrels [10].

A relevant issue in any long-term exercise programme is its adherence. This is especially challenging for people with PD since they face several barriers. Maintaining long-term adherence to any exercise program is challenging for people with PD due to the existence of several barriers of exercise that they must face. For instance, to motivate this population to exercise, it is important that they expect to derive benefit from its practice [11]. In this regard, there is evidence that Pilates is an effective form of exercise for patients with multiple sclerosis, a neurological disorder that impairs motor and non-motor function [12], as in the case of PD.

Other barriers to exercise include lack of time and transportation difficulties [11]. In relation to this, Pilates is an activity that can be performed independently by the patient after a short period of training at home. Finally, the novelty of the programme has been regarded as an important fact that improves adherence to exercise by people with PD [13]. Therefore, Pilates can be considered an exercise that can be developed without supervision or the need to register or move to a specific facility. Furthermore, patients may feel that Pilates can help with controlling the symptoms of their condition and thus, feel attracted to the innovative approach of Pilates.

In the light of all this, the purpose of this systematic review was to identify and critically analyze existing scientific evidence on the feasibility and benefits of Pilates for PD.

## 2. Materials and Methods

### 2.1. Data Sources and Searches

**Figure 1:**
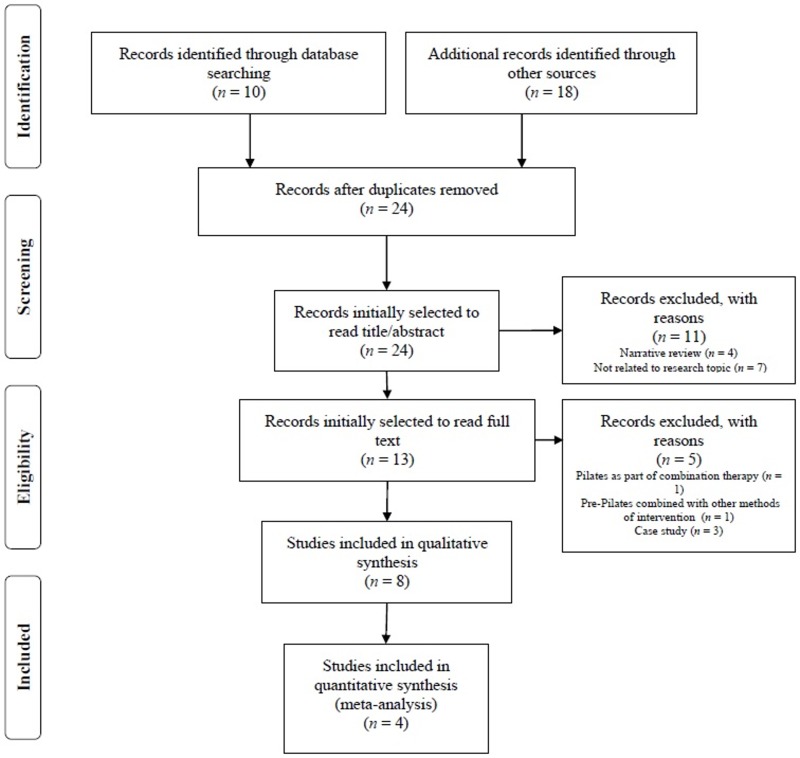
Flow chart of the review process.

This systematic review was conducted in accordance with the Preferred Reporting Items for Systematic Review and Meta-Analysis Protocols (PRISMA-P) checklist [14]. The systematic review process is included in Figure 1. The selected search strategy and methods of analysis were registered in the PROSPERO database (ref: CRD42019131779). An electronic search was performed in PubMed, PEDro, Scopus and SPORTDiscus databases during March 2019.

Based on the Population, Intervention, Comparison and Outcome (PICO) strategy and following the recommendations of the Cochrane Handbook for Systematic Reviews of Interventions [15], only the terms regarding the population and the intervention were used in a combination of MeSH and free-text terms. Therefore, the keywords used were “Pilates” and “Parkinson’s disease”, with the Boolean operator “AND” between terms (See Appendix A). Additional searches of relevant references within included articles and existing systematic reviews were performed manually. The titles of all the studies found in every database and after checking the reference list of the included papers were registered on an Excel worksheet in order to detect duplicated publications.

### 2.2. Eligibility Criteria

Randomized and non-randomized controlled studies were included if they satisfied the following criteria: (a) reporting the original data for any type of Pilates intervention; (b) the target population was people diagnosed with PD; (c) reporting at least one outcome measure related to the main PD symptoms. Studies were excluded if they: (a) included people with several neurological impairments, unless separate data were available for the PD subgroup; (b) did not describe the physical training programme carried out; (c) analyzed the effects of an isolated exercise session; (d) used Pilates in combination with other rehabilitative therapies; (e) were written in a language other than Spanish, Portuguese or English.

### 2.3. Data Extraction

Information on the study design, sample characteristics, Pilates programme, study variables, assessment tools, and relevant results were extracted from the original reports by one researcher and checked by a second investigator. The data extraction procedure was not blind, as the names of the authors of the selected studies and the title of the journals in which they were published were identifiable. Missing data were obtained from the study authors whenever possible.

### 2.4. Quality Assessment

The methodological quality of randomized controlled trials (RCTs) was determined by using the PEDro scale [16]. The total score obtained in this scale served to differentiate the quality of the investigations between “high” (6 or more points) or “low” (5 or fewer points) [17]. For the non-RCT studies, the “Quality Assessment Tool for Before-After (Pre-Post) Studies with No Control Group” (NIH) [18] was applied. This scale includes 12 questions from which a total score is obtained. The quality of each study was classified as “poor”, “fair” or “good”. Two authors of the present study independently administered both scales. The authors next compared and contrasted the scores provided by each for all the studies. In cases of score disagreement, a third author was asked to independently score the studies in question in order to reach a consensus.

### 2.5. Data Synthesis

Only RCTs were included in the meta-analysis. When at least two RCTs reported baseline and post-treatment data on homogeneous outcome measures, a meta-analysis was intended to be carried out [19]. For this purpose, pooled effect sizes were estimated according to a fixed effect model and a random-effects model. Hedges’ *g* and the 95% confidence intervals (95% CI) were calculated to assess the difference in change between the Pilates intervention and comparison group using the baseline and post-treatment sample sizes, means (M), and standard deviations (SD) for the selected variables. Heterogeneity was assessed using I^2^ statistics. Hedges’ *g* was considered significant when their 95% CI excluded zero, while pooled Hedges’ *g* values were interpreted according to Cohen [20], whereby effects were considered small (0.2), medium (0.5), and large (0.8). Positive effect size estimates were indicative of the Pilates intervention having a positive post-treatment effect on the corresponding outcome variable, with negative values favouring the comparison group. The significance level was *p* < 0.05 for all analyses. Data were analyzed using Stata Software version 15.1 (Stata Corporation, College Station, TX, USA) [21].

## 3. Results

### 3.1. Design and Samples

Out of the 28 studies initially obtained, eight were finally selected, including four RCTs [22,23,24,25] and four non-RCT studies [26,27,28,29] (Figure 1). The four RCTs were combined in the meta-analysis. Inclusion was based on pre and post comparable information regarding the effects of Pilates on functional mobility and lower limb strength for both the comparison and Pilates groups.

### 3.2. Quality Assessment

The methodological quality of the RCTs was considered “poor” in the four studies analysed [22,23,24,25]. The non-RCT studies showed methodological quality ranging from “poor“ [26] to “fair“ [27,28,29]. (Table 1).

### 3.3. Interventions

The main characteristics of the proposed Pilates interventions are shown in Table 2. With the exception of one study, all the interventions were based on the performance of mat Pilates, including exercises assisted by materials such as an elastic band or Swiss ball, except for one study that used machines, specifically a reformer [29]. The duration of the programmes ranged from 6 to 12 weeks and included 60-min sessions conducted 2–3 times per week. The interventions focused primarily on improving muscle strength and range of motion, particularly the core and lower limbs. For the duration of the programmes, the difficulty, duration, and repetitions of the exercise were gradually increased according to the conditions of the participants.

In a total of three studies, Pilates was compared with other training interventions based on walking [23,24] or calisthenics [22]. In one study, participants in the Pilates group were asked to exercise at home to maintain continuity of treatment [25].

### 3.4. Compliance and Adverse Events

During the interventions, a loss to follow-up of 6.25% was reported in one investigation [24], and 7.69% for another [22]. All participants completed the intervention in three studies [26,27,29], whereas the remaining three studies failed to report attrition rates [23,25,28]. On the other hand, only two studies recorded adherence, both of which were higher than 80% [22,27].

No adverse events were observed in two studies [22,29]. An additional two studies reported only minor adverse effects in the intervention conditions, including one participant suffering mild side effects such as fatigue, muscle pain, and cramps [24], and another participant experiencing mild dizziness [27]. The remaining four studies failed to report the presence or absence of any adverse events [23,25,26,28].

### 3.5. Main Outcomes

#### 3.5.1. Physical Fitness

Half of the studies analyzing the effects of Pilates indicated significant improvements in variables related to physical fitness. Specifically, participants showed positive changes in lower limb strength [22,23,28], upper limb strength [27,28], lower limb flexibility [27,28], lumbo-pelvic stability [23], aerobic endurance [27], and body mass index [22]. Interestingly, one of the non-RCT studies indicated benefits of Pilates on the general physical fitness of the participants [26].

#### 3.5.2. Balance

Three RCTs found significantly larger improvements in the Pilates treatment than in comparison conditions [23,24,25]. Four studies comparing balance before and after Pilates treatments also found significant intragroup improvements [23,24,25,29]. In addition, two studies [25,29] included balance confidence during daily activities as an outcome measure. Pandya et al. [25] indicated significant improvement after the Pilates programme, while Johnson et al. [29] found only marginal benefits.

#### 3.5.3. Functional Mobility

Statistically significant changes were observed in three of the five investigations that analyzed functional mobility, measured by the Timed Up and Go (TUG) test [22,24,25,28,29]. In three studies, the practice of Pilates resulted in significant improvements in the time required to complete the test [22,24,26], while Johnson et al. [29] demonstrated significant benefits in cadence in terms of steps/minute.

#### 3.5.4. Quality of Life

Out of the two studies [27,28] that analyzed the impact of Pilates on patients’ reported quality of life, only one reported significant benefits [27].

#### 3.5.5. Results of the Meta-Analysis

**Figure 2:**
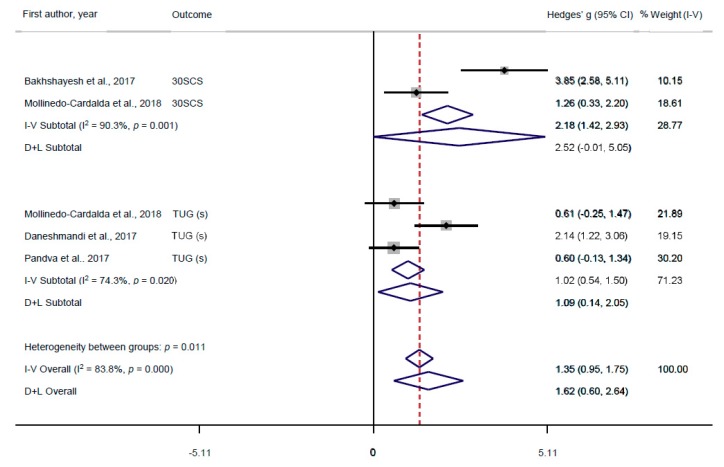
Forest plot of effect size estimates on Timed Up and Go (TUG) and 30-Second Chair Stand (30SCS) tests in Pilates interventions versus comparison groups. The squares represent the effect size estimate (Hedges’ *g*) and horizontal lines represent the confidence intervals (CI) for each RCT. The diamonds represent the effect size estimates for subgroups and the overall effect. The vertical line represents the null hypothesis (Hedges’ *g* = 0). The vertical dotted line represents the overall mean difference from all RCTs. A positive Hedges’ *g* is indicative of the Pilates intervention having a greater effect on TUG and 30SCS scores than the comparison group.

Data from a total of 112 (57 intervention and 55 comparison) participants across four RCTs were included in the meta-analysis to compare the effects of intervention groups with comparison groups. Two RCTs reported outcome data for the 30-Second Chair Stand (30SCS) test [22,23], and three RCTs reported outcome data for the TUG test [22,24,25]. An Egger’s regression test [30] on the TUG (s) outcome indicated no presence of publication bias (bias = −12.31, *p* = 0.468). Publication bias could not be tested on the 30SCS outcome as it only contained two effect size estimates. Both variables were related to lower-body functioning, which were reported as Hedges’ *g* for each variable and the pooled estimates. Please see Figure 2 for complete analyses.

When assessing the impact of Pilates programmes on the lower-body functioning of individuals suffering from PD, significant heterogeneity between studies was observed. Pooled effect sizes from two RCTs reported a significant difference in 30SCS, with a pooled Hedges’ *g* = 2.52 (95% CI = −0.01, 5.05; *p* < 0.001); I^2^ = 90.3%; *p* < 0.01, indicating that Pilates had a greater improvement on the 30SCS test than the comparison group. Notably, the lower CI included negative values, however these values were negligible. Similarly, pooled effect sizes from three RCTs reported a significant difference in TUG, with a pooled Hedges’ *g* = 1.09 (95% CI = 0.14, 2.05; *p* < 0.001); I^2^ = 74.3%; *p* < 0.05, indicating that Pilates had a greater improvement on TUG test than the comparison group. The overall pooled results showed a significant improvement in global lower-body functioning, Hedges’ *g* = 1.62 (95% CI = 0.60, 2.64; *p* < 0.001); I^2^ = 83.8%; *p* < 0.001.

## 4. Discussion

In this study the existing scientific evidence on the effectiveness of Pilates as a rehabilitation strategy in patients with PD was examined and critically reviewed. For this purpose, the search was designed to find the largest number of studies, regardless of their design. This was due to the fact that, when examining the existing scientific evidence on a physical therapy and the number of RCTs found is small, it is difficult to draw consistent conclusions. In addition, non-RCT studies may also provide relevant information on proposed interventions, such as feasibility, dosage, in terms of types of exercises, repetitions, series and rests, and the existence of adverse effects [12]. Knowing all these details can assist neurologists and medical professionals in deciding whether to recommend Pilates to patients as a form of physical therapy.

In addition to the small number of reports found using the search strategy mentioned, half of these used a single-subject design with small sample sizes. In addition, the four RCTs analyzed showed a poor methodological quality, mostly due to a lack of concealed allocation and blinding. Considering these circumstances, the findings presented herein should be considered as preliminary evidence and therefore, should be interpreted with caution.

Physical exercise is known to have beneficial effects on PD patients and they are more motivated to practice it if recommended by their physician or neurologist [31]. However, it appears that neurologists do not usually prescribe physical exercise to PD patients in their early stages [32]. The present review affords some relevant information that may contribute to increase the current knowledge on the viability of Pilates as part of an integral rehabilitation strategy in patients with PD.

Before prescribing any kind of physical therapy, its feasibility should be evaluated. Only one of the studies analyzed in this review addressed this issue and concluded that Pilates was a feasible therapy for people with PD. Although not all the investigations provided accurate data in this regard and judging from the findings of those studies in which information related to adherence, drop-outs and adverse effects were reported, it seems that Pilates can be a feasible rehabilitation approach in this population.

It has been reported that the self-perceived benefit of the training programme is a major reason why PD patients decide to start exercising [32]. In the present review, the practice of Pilates seemed to be effective in improving physical fitness, an aspect to be taken into account in the design of physical exercise programmes for this population [33]. In addition, it appears to contribute to the improvement of balance problems, as three of the studies that analyzed intra and intergroup changes in this variable reported significant improvements. This finding is worth mentioning given the importance of physical rehabilitation to improve balance, contributing to the stability of the gait pattern and reducing the risk of falls [34]. Nevertheless, given the low number of studies found and their characteristic, more solid conclusions cannot be made. This indicates that patients’ expectations regarding the impact of Pilates on their fitness and balance levels should, at most, be modest. It should also be noted that only two studies integrated quality of life as a study variable and only one of them found improvements in it. This indicates the need for further research in this regard.

The importance for Pilates interventions to be replicated in future studies or by practitioners should not be overlooked. Presently, Pilates is regarded a popular therapy in mainstream exercise and rehabilitation. As a consequence, a variety of Pilates instructors have emerged, including ex-dancers and fitness professionals, who usually perform Pilates-inspired programmes created to capitalize on the popularity of the method [10]. However, the proposed exercises are not Pilates techniques at all. To avoid this malpractice, proper Pilates exercises based on traditional application of physical therapy principles focused on the maintenance of an optimal posture, the contraction of truncal muscles and proper breathing during the exercises, should be performed under the initial supervision of a certified instructor or physiotherapist. All the studies selected in this review included well-known Pilates exercises that, in some cases, were adapted to the characteristics of the participants, also known as pre-Pilates. However, not all the interventions were described in detail, which restricts the possibility of replicating some of the training programmes proposed. Moreover, only three studies specified that a physiotherapist or a qualified Pilates instructor led the programme. This fact could be misleading, since Pilates is considered a well-stablished physical therapy method, and only specialists in this field should be allowed to design and instruct these kinds of programmes, especially those aimed at restoring function in people with PD.

Despite the small number of RCTs found, all included relevant information on the effects of Pilates on the functionality of the lower limbs, which could also be meta-analyzed. Judging from the scarce available literature, there is evidence indicating that their practice could be more effective than more traditional training programmes. This is a factor to consider in the prescription of Pilates to patients with PD, and it is expected that increases in the strength of the lower limbs and in functional autonomy will translate into improvements in their functional mobility, allowing them to face daily activities with greater safety and efficiency [35].

The study presented herein provides preliminary evidence indicating that Pilates may be prescribed to patients with PD as a feasible therapy that could lead to positive changes in their physical fitness, balance and functionality of their lower limbs. However, the absence of studies on the benefits of Pilates on other important motor and non-motor symptoms of the disease, for example anxiety, depression, or apathy, does not allow us to conclude that it is equally or more effective than other physical rehabilitation modalities for these patients.

When interpreting the results of a systematic review, a number of limitations related to both the characteristics of the selected investigations and the methodological design of the study review should be taken into account. Regarding to the first aspect, the reduced number of studies found, the small sample size of some of them, and their generally low methodological quality indicate the need for more research to consolidate the existing scientific evidence. In addition, the participants included in the studies showed mild-to-moderate PD according to stages I–III on the Hoehn & Yahr scale [36]. Consequently, the findings do not apply to patients in later stages of the disease. As far as the design of the present work is concerned, restricting the languages used for the search, not having reviewed the grey literature and the possible existence of publication bias were the main methodological weaknesses to be recognized.

## 5. Conclusions

Pilates can be safely prescribed for people with mild-to-moderate PD. Preliminary evidence indicates that its practice could have a positive impact on fitness, balance and physical function. Its benefits on lower-body function appear to be superior to those of other conventional exercises. Future randomized studies with greater samples are needed to confirm these observations.

## Figures and Tables

**Table 1:** Quality assessment.

**PEDro Scale**	**1**	**2**	**3**	**4**	**5**	**6**		**7**	**8**	**9**	**10**	**11**	**Total**
Mollinedo-Cardalda et al., 2018 [22]	Y*	Y	N	Y	N	N		N	N	N	Y	Y	4/10
Bakhshayesh et al., 2017 [23]	Y*	Y	N	Y	N	N		N	Y	N	Y	Y	5/10
Daneshmandi et al., 2017 [24]	Y*	Y	N	Y	N	N		N	Y	N	Y	Y	5/10
Pandya et al., 2017 [25]	Y*	N	N	Y	N	N		N	Y	Y	Y	Y	5/10
**NIH Pre-Post Tool**	**1**	**2**	**3**	**4**	**5**	**6**	**7**	**8**	**9**	**10**	**11**	**12**	**Total**
Do Carmo et al., 2018 [26]	Y	Y	Y	NR	N	Y	Y	NR	Y	N	N	* NA	6/11
Cancela et al., 2018 [27]	Y	Y	Y	Y	N	Y	Y	N	Y	Y	N	* NA	8/11
Hartmann et al., 2014 [28]	Y	Y	Y	NR	N	Y	Y	NR	Y	Y	N	* NA	7/11
Johnson et al., 2013 [29]	Y	Y	Y	NR	N	Y	Y	NR	Y	Y	N	* NA	7/11

Note. Y, yes; N, no; NA, not applicable; NR, not reported. * Not included in total score.

**Table 2:** Characteristics of the studies included in the systematic review.

First Author, Year	Study Design	Participants	Intervention and Comparison Groups	Outcomes of Interest	Results
Mollinedo-Cardalda et al., 2018 [22]	RCT	IG: *n* = 12 (62.85 ± 9.75 years) CG: *n* = 10 (66 ± 13.14 years) PD Criteria: Idiopathic PD (stage I-III on the Hoehn and Yahr scale).	Length: 12 weeks IG: Four experts in physical activity and neurodegenerative diseases from university designed and implemented 24 sessions at a rate of two 60-min sessions per week, including mat Pilates exercises adapted for PD populations using Medium-Resistant TheraBand^®^ as well as 0.5 kg ankle and/or wristbands, with 7 exercises distributed in 3 sets of 8 repetitions at a rating of 7 on the Modified Borg Rating of Perceived Exertion. CG: Calisthenics that combined aerobic exercises, such as different varieties of marching, with strength, flexibility, joint mobility and coordination tasks, leaving 10 min for warm-up focused on joint mobility exercises and 5 min for cool-down and muscle group stretching.	30SCS (reps) TUG (s) Unified Parkinson’s Disease Rating Scale (UPDRS) III (score) Five Times Sit to Stand (FTSS) test (s)	Attrition: 4 (IG = 1, CG = 3) Adherence: 80.21% Adverse Events: None Intergroup Differences (Post-Test): Improvements favored the IG more than the CG in: 30SCS (reps): 23.8 ± 6.4 vs. 12.3 ± 5.1; *p* < 0.05 TUG (s): 7.8 ± 2.8 vs. 9.2 ± 2.5; *p* < 0.05 FTSS test (s): 5.78 ± 1.5 vs. 9.1 ± 4.1; *p* < 0.05 Intragroup Differences (Pre-Post): Improvements for IG in: 30SCS (reps): 17.8 ± 6.4 vs. 23.8 ± 6.4; *p* < 0.001 TUG (s): 9.4 ± 2.4 vs. 7.8 ± 2.8; *p* < 0.001 FTSS test (s): 9.0 ± 4.0 vs. 5.8 ± 1.5; *p* < 0.05
Bakhshayesh et al., 2017 [23]	RCT	IG: *n* = 15 (57 ± 6.24 years) CG: *n* = 15 (58.31 ± 7.37 years) PD Criteria: Moderate idiopathic PD (stage II-III on the Hoehn and Yahr scale); diagnosis at least three years before; the ability to independently stand and walk; not using balance and walking aids; having a history of balance and walking disorders owing to PD.	Length: 8 weeks IG: A Pilates instructor conducted 24 sessions at a rate of three 60-min sessions per week, including 10-min warm-up stretching and mobility exercises for the upper and lower extremity and the trunk, then 45 min performing 10 selected Pilates exercises aimed at increasing the strength of core muscles, range of motion and strength of lower limb, and ending with 5 min of recovery exercises including stretching and relaxation exercises. CG: Progressive walking from 10 min in the first week to 30 min at the end of the training period.	30SCS (reps) Fullerton Advanced Balance (FAB) scale (score) Step-Down (S-D) test (reps) Trunk Flexion Endurance (TFE) test (s) Trunk Extension Endurance (TEE) test (s) Trunk Lateral Flexion Endurance (TLFE) test (s)	Attrition: NR Adherence: NR Adverse Events: NR Intergroup Differences (Post-Test): Improvements favored the IG more than the CG in: 30SCS (reps): *M*_diff_ = 12.5 ± 1.6; *p* < 0.001 FAB scale (score): *M*_diff_ = 25.4 ± 1.5; *p* < 0.001 S-D test (reps): *M*_diff_ = 38.1 ± 2.8; *p* < 0.001 TFE test (s): *M*_diff_ = 58.9 ± 4.8; *p* < 0.001 TEE test (s): *M*_diff_ = 30.4 ± 1.9; *p* < 0.001 TLFE test (s): *M*_diff_ = 47.5 ± 2.2; *p* < 0.001 Intragroup Differences (Pre-Post): Improvements for IG in: 30SCS (reps): *M*_diff_ = 14.1 ± 3.5; *p* < 0.001 FAB scale (score): *M*_diff_ = 25.7 ± 3.7; *p* < 0.001 S-D test (reps): *M*_diff_ = 38.1 ± 10.6; *p* < 0.001 TFE test (s): *M*_diff_ = 58.7 ± 19.5; *p* < 0.001 TEE test (s): *M*_diff_ = 29.8 ± 5.9; *p* < 0.001 TLFE test (s): *M*_diff_ = 47.4 ± 8.5; *p* < 0.001
Daneshmandi et al., 2017 [24]	RCT	IG: *n* = 15 (57 ± 6.24 years) CG: *n* = 15 (58.31 ± 7.37 years) PD Criteria: Moderate idiopathic PD (levels II-III on the Hoehn and Yahr scale); passage of at least 3 years after the disease diagnosis; the ability to stand and walk independently; not using assistive devices for keeping balance and walking; the history of balancing and walking disorders due to PD.	Length: 8 weeks IG: A Pilates trainer conducted 24 sessions at a rate of three 60-min sessions per week, including 10 min of warm-up exercises, 45 min of 10 selected Pilates exercises with the purpose of increasing the strength of body core muscles and lower limb joints’ range of motion, and finishing with 5 min of cool down exercises. Exercises began with 6–8 repetitions and without any exercise resistance, then volume was regularly increased. CG: Walking during training period.	TUG (s) Fullerton Advanced Balance (FAB) scale (score)	Attrition: 2 (IG = 1, CG = 1) Adherence: NR Adverse Events: Fatigue, muscle pain, and cramps (*n* = 1) Intergroup Differences (Post-Test): Improvements favored the IG more than the CG in: TUG (s): *M*_diff_ = −1.0 ± 1.6; *p* < 0.001 FAB scale (score): *M*_diff_ = 25.4 ± 1.5; *p* < 0.001 Intragroup Differences (Pre-Post): Improvements for IG in: TUG (s): 17.6 ± 6.1 vs. 8.7 ± 2.6; *p* < 0.001 FAB scale (score): 7.0 ± 2.3 vs. 32.7 ± 5.2; *p* < 0.001
Pandya et al., 2017 [25]	RCT	IG: *n* = 15 (58.46 years) CG: *n* = 15 (58 years) PD Criteria: Idiopathic PD diagnosed by a neurologist (stage I-III on the Hoehn and Yahr scale); diagnosed before a year or more and were on stable pharmacological treatment; no severe dyskinesia and/or motor fluctuations.	Length: 7 weeks IG: 21 sessions at a rate of three 60-min sessions per week, including supervised Pilates training and exercises to be carried out at home to maintain continuity in the treatment. CG: 21 sessions at a rate of three 60-min sessions per week, including conventional therapy.	TUG (s) Berg Balance Scale (BBS) (score) Activity-specific Balance Confidence (ABC) scale (score)	Attrition: NR Adherence: NR Adverse Events: NR Intergroup Differences (Post-Test): Improvements favored the IG more than the CG in: TUG (s): 23.5 ± 4.2 to 18.0 ± 8.8 vs. 27.8 ± 3.2 to 26.5 ± 3.6; *p* < 0.01 BBS (score): 38.0 ± 3.2 to 42.1 ± 5.6 vs. 35.7 ± 4.6 to 37.1 ± 5.1; *p* < 0.05 ABC scale (score): 35.0 ± 9.5 to 48.7 ± 12.9 vs. 32.9 ± 10.0 to 34.8 ± 9.7; *p* < 0.01 Intragroup Differences (Pre-Post): Improvements for IG in: TUG (s): 23.5 ± 4.2 vs. 18.0 ± 8.8; *p* < 0.01 BBS (score): 38.0 ± 3.2 vs. 42.1 ± 5.6; *p* < 0.001 ABC scale (score): 35.0 ± 9.5 vs. 48.7 ± 12.9; *p* < 0.01
Do Carmo et al., 2017 [26]	Single-subject	Sample: *n* = 4 (73.5 ± 8.6 years) PD Criteria: Diagnosis of PD (stage 1.5–2.5 on the Hoehn and Yahr scale).	Length: 10 weeks IG: A physiotherapist with training in the Pilates method carried out 30 sessions at a rate of three 60-min sessions per week, including pre-Pilates, mat Pilates, balance and gait training, and diaphragmatic respiratory training.	30SCS (n) Chair Sit-and-Reach (CSR) test (cm) Back-Scratch test (cm) 2-Minute Step test (steps) 8-Foot Up and Go test (s) Arm Curl test (reps)	Attrition: None Adherence: NR Adverse Events: NR Intragroup Patterns (Pre-Post): No significance tests were used. In general, post-intervention improvements were observed for the CSR test, Back-Scratch test, 8-Foot Up and Go test, and Arm Curl test.
Cancela et al., 2018 [27]	Single-subject	Sample: *n* = 16 (69.13 ± 8.23 years) PD Criteria: Idiopathic PD with a favorable response to L-Dopa (stage I-III on the Hoehn and Yahr scale); clinical stability with no changes in antiparkinson therapy three months prior to the start of the programme; able to ambulate independently.	Length: 12 weeks IG: A Pilates-certified physical therapist with 2 years MP experience lead 24 sessions at a rate of two 60-min sessions per week, including mat Pilates aimed at improving mobility, strength, and coordination by performing breathing, postural stability and body awareness exercises. One session was based on mat Pilates exercises which are traditionally performed while lying on the floor and the other session was focused on the performance of modified mat Pilates exercises adapted to standing and seated positions to improve posture and movement control of the activity.	30SCS (n) Chair Sit-and-Reach (CSR) test (cm) Back-Scratch test (cm) 2-Minute Step test (steps) 8-Foot Up and Go test (s) Parkinson’s Disease Questionnaire (PDQ) (score) Arm Curl (AC) test (reps)	Attrition: None Adherence: 81.25% Adverse Events: Mild dizziness (*n* = 1) Intragroup Differences (Pre-Post): Improvements for IG in: 30SCS (n): 12.5 ± 3.3 vs. 16.9 ± 4.1; *p* < 0.001 CSR test (cm): 0.5 ± 9.1 vs. 3.9 ± 6.6; *p* < 0.05 2-Minute Step test (steps): 65.3 ± 21.2 vs. 84.3 ± 19.3; *p* < 0.05 PDQ (score): 14.1 ± 8.0 vs. 11.4 ± 7.7; *p* < 0.05 AC test (reps): 13.9 ± 3.6 vs. 22.2 ± 3.5; *p* < 0.001
Hartmann et al., 2014 [28]	Single-subject	Sample: *n* = 7 (62 years) PD Criteria: Medical diagnosis of PD; walking without bracing or prosthesis.	Length: 10 weeks IG: 20 sessions at a rate of two 60-min sessions per week, including mat Pilates exercises performed in a series of ten repetition, in a university setting.	30SCS (n) TUG (s) Chair Sit-and-Reach (CSR) test (cm) Parkinson’s Disease Questionnaire (PDQ) (score) Turnaround time (360°) 30-S Elbow Flexion (30-s EF) test (reps) One-legged stance test (s)	Attrition: NR Adherence: NR Adverse Events: NR Intragroup Differences (Pre-Post): Improvements for IG in: 30SCS (n): *M*_diff_ = 2.6 ± 2.0; *p* < 0.05 CSR test (cm): *M*_diff_ = 7.2 ± 6.5; *p* < 0.05 30-s EF test (reps): *M*_diff_ = 2.7 ± 2.5; *p* < 0.05
Johnson et al., 2013 [29]	Single-subject	Sample: *n* = 10 (67.6 ± 8.9 years) PD Criteria: Idiopathic PD (stage I-III on the Hoehn and Yahr scale); were on a stable drug regimen; no severe dyskinesia and/or motor fluctuations.	Length: 6 weeks IG: A qualified Pilates instructor and an assistant lead 12 sessions at a rate of two 60-min sessions per week, including a combination of plinth exercises, gym-ball exercises, stepping exercises, and exercises on a Pilates reformer, with the degree of difficulty increasing progressively.	TUG (s) Berg Balance Scale (BBS) (score) Activity-specific Balance Confidence (ABC) scale (%) Unified Parkinson’s Disease Rating Scale (UPDRS) (score) Schwab and England Scale (SES) 5-m walk (s) Static posturography Dynamic posturography	Attrition: None Adherence: NR Adverse Events: None Intragroup Differences (Pre-Post): Improvements for IG in: BBS (score): 47.1 ± 2.0 vs. 50.4 ± 1.5; *p* < 0.05 5-m walk (s): 6.3 ± 0.4 vs. 5.5 ± 0.3; *p* < 0.05

Note. Statistics are reported as means ± standard deviations unless otherwise specified. *M*_diff_, mean difference; NR, not reported; PD, Parkinson’s disease; IG, intervention group; CG, comparison group; 30SCS (reps), 30-Second Chair Stand test (number of times the participant can sit down and stand up from a chair in 30 s); TUG (s), Timed Up and Go test (seconds).

## Appendix A. Search strategy.

**PubMed search**		
**Keywords**	**Pilates, Parkinson’s disease**	
Descriptors (thesaurus)	None	
Applied Filters	None	
Search equation, nº of results obtained, time period of publication	Pilates [All Fields] AND (“Parkinson disease”[MeSH Terms] OR (“Parkinson”[All Fields] AND “disease”[All Fields]) OR “Parkinson disease”[All Fields] OR (“Parkinson’s”[All Fields] AND “disease”[All Fields]) OR “Parkinson’s disease”[All Fields])	3
		1972–2019
**Pedro search**		
Keywords	Pilates, Parkinson’s disease	
Descriptors (thesaurus)	None	
Applied Filters	None	
Search equation, nº of results obtained, time period of publication	Pilates [All Fields] AND (“Parkinson’s disease”[All Fields])	1
		1999–2019
**SCOPUS search**		
Keywords	Pilates, Parkinson’s disease	
Descriptors (thesaurus)	None	
Applied Filters	None	
Search equation, nº of results obtained, time period of publication	TITLE-ABS-KEY (Pilates AND “Parkinson’s Disease”)	6
		1978–2019
**SPORTDiscus search**		
Keywords	Pilates, Parkinson’s disease	
Descriptors (thesaurus)	None	
Applied Filters	None	
Search equation, nº of results obtained, time period of publication	Pilates AND Parkinson’s disease	3
		1970–2019
